# Development of a docetaxel micellar formulation using poly(ethylene glycol)–polylactide–poly(ethylene glycol) (PEG–PLA–PEG) with successful reconstitution for tumor targeted drug delivery

**DOI:** 10.1080/10717544.2018.1477865

**Published:** 2018-06-05

**Authors:** Taehoon Sim, Jae Eun Kim, Ngoc Ha Hoang, Jin Kook Kang, Chaemin Lim, Dong Shik Kim, Eun Seong Lee, Yu Seok Youn, Han-Gon Choi, Hyo-Kyung Han, Kwon-Yeon Weon, Kyung Taek Oh

**Affiliations:** a College of Pharmacy, Chung-Ang University, Seoul, Republic of Korea;; b College of Pharmacy & Institute of Pharmaceutical Science and Technology, Hanyang University, Ansan, Republic of Korea;; c Department of Biotechnology, The Catholic University of Korea, Bucheon, Republic of Korea;; d School of Pharmacy, SungKyunKwan University, Suwon City, Republic of Korea;; e College of Pharmacy, Dongguk University-Seoul, Goyang, Republic of Korea;; f College of Pharmacy, Catholic University of Daegu, Gyeongsan-si, Republic of Korea

**Keywords:** Nanomedicine, lyophilization, cancer, reconstitution, polymeric micelle

## Abstract

Docetaxel (DTX)-loaded polymeric micelles (DTBM) were formulated using the triblock copolymer, poly(ethylene glycol)–polylactide–poly(ethylene glycol) (PEG–PLA–PEG), to comprehensively study their pharmaceutical application as anticancer nanomedicine. DTBM showed a stable formulation of anticancer nanomedicine that could be reconstituted after lyophilization (DTBM-R) in the presence of PEG 2000 and D-mannitol (Man) as surfactant and protectant, respectively. DTBM-R showed a particle size less than 150 nm and greater than 90% of DTX recovery after reconstitution. The robustly formed micelles might minimize systemic toxicity due to their sustained drug release and also maximize antitumor efficacy through increased accumulation and release of DTX from the micelles. From the pharmaceutical development point of view, DTBM-R showing successful reconstitution could be considered as a potent nanomedicine for tumor treatment.

## Introduction

Recently, various nanocarriers have shown potential to overcome the limitations of drugs that exhibit high efficacy but low bioavailability due to poor solubility through tumor-targeted drug delivery (Peer et al., 2007; Wicki et al., 2015; Choi et al., 2016; Shi et al., 2017; Choi & Han, 2018). There has been great interest in polymeric micelles based on amphiphilic block copolymers as nanocarriers for anticancer therapeutics (Kataoka et al., 2001; Gaucher et al., 2005). In aqueous environments, amphiphilic block copolymers with a higher concentration than the critical micelle concentration (CMC) form a core–shell structure as a thermodynamically stable state by self-assembly. The hydrophobic core plays a crucial role in the incorporation of hydrophobic anticancer agents (Adams et al., 2003). Furthermore, the properties of block copolymers can be optimized by altering their chemical compositions for successful pharmaceutical formulation and drug delivery (Allen et al., 1999; Rösler et al., 2001; Torchilin, 2007).

In previous reports, poly(ethylene glycol)–polylactide–poly(ethylene glycol) (PEG–PLA–PEG) was developed for tumor-targeted drug delivery (Song et al., 2016; Hoang et al., 2017a,b). As a biocompatible water-soluble polymer, PEG has widely been utilized as a hydrophilic block because of its outstanding water solubility, chain mobility, nontoxicity, and non-immunogenicity (Yamaoka et al., 1994). Poly(lactide) (PLA) is a biocompatible and biodegradable polymer with low immunogenicity and favorable mechanical properties for pharmaceutical and biomedical applications (Nampoothiri et al., 2010; Rasal et al., 2010; Oh, 2011). The triblock copolymer was synthesized by Steglich esterification and showed promising properties including high stability, high drug loading efficiency, and successful reconstitution, compared to PEG–PLA diblock copolymer (Song et al., 2016). In addition, PEG–PLA–PEG showed potential as an anticancer nanomedicine for metastatic breast cancer by tumor-targeted drug delivery with high therapeutic efficacy (Hoang et al., 2017a,b).

From a pharmaceutical point of view, micellar formulations in aqueous solution may result in the degradation of incorporated drugs and the excipients including polymers, during storage. The stability issue might be overcome by using a powder state of micellar formulation that could be reconstituted to a colloidal system by simply adding excipients such as surfactants and protectants. Furthermore, this is a practical approach with respect to *in vivo* applications, large-scale preparation of the product, and drug stability (Abdelwahed et al., 2006; Oh et al., 2008, Fonte et al., 2016).

In this study, docetaxel (DTX) was loaded into triblock copolymer micelles using PEG–PLA–PEG (DTBM) and the pharmaceutical application of the nanomedicine was comprehensively studied and compared with a commercial product, Nanoxel M, composed of docetaxel loaded polymeric micelle. Preparation of DTBM, physicochemical characterization, optimization of a DTX micellar formulation with successful reconstitution (DTBM-R), *in vitro* study, and *in vivo* therapeutic evaluation were conducted to demonstrate the potential of the polymeric nanocarrier using PEG–PLA–PEG from the point of view of pharmaceutical development.

## Materials and methods

### Materials

Poly(ethylene glycol) 2000 (MW 2 kDa, PEG 2K), dimethylformamide (DMF), PVA, Pluronic F68, PEG 5000 (PEG 5K), PEG 400, tween 80, trehalose, sucrose, lactose, glycine, and D-mannitol (Man) were purchased from Sigma-Aldrich (St. Louis, MO). Tetrahydrofuran (THF), toluene, acetone, and dichloromethane (DCM) were purchased from Honeywell Burdick & Jackson^®^ (Muskegon, MI). Docetaxel (DTX) and the commercial DTX formulation, Nanoxel M were purchased from Samyang Biopharmaceuticals Corp. (Seongnam-si, Gyeonggi-do, Korea). Diethyl ether and hexane were purchased from Samchun Chemical (Hunt Valley, MD). Cyanine 5.5 amine (Cy5.5 amine) was purchased from Lumiprobe (Waltham, MA). KB cells were obtained from Korean Cell Line Bank (Jongno-gu, Seoul, Korea). RPMI 1640 medium, DPBS, penicillin–streptomycin solution, trypsin-EDTA solution, and fetal bovine serum (FBS) were purchased from Welgene (Gyeongsan-si, Gyeongsangbuk-do, Korea). Cell Counting Kit-8 (CCK-8) was purchased from Dojindo Molecular Technologies, Inc. (Rockville, MD). PEG–PLA–PEG triblock copolymers were synthesized using procedures described previously (Hoang et al., 2016, 2017b; Song et al., 2016). Detailed synthesis and characterization of PEG–PLA–PEG are described in the Supporting Information.

### Methods

#### Preparation of drug-loaded micelles

For the preparation of DTX-loaded micelles, 0.5 mg DTX was mixed with 5 mg of triblock copolymer in 7 ml of a mixture of DMF and distilled water (DMF:DW = 2:5) and dialyzed (MWCO 3.5 kDa, REPLIGEN, Waltham, MA, USA) against distilled water for 24 h. The solutions were centrifuged at 5000 rpm for 5 min to precipitate unloaded drug. Supernatant containing drug-loaded micelles was collected and analyzed.

#### Measurement of DTX concentration by HPLC

The DTX concentrations of the micelles and other samples were analyzed using a high-performance liquid chromatography system (HPLC, Agilent 1200 series, Agilent Tech., CA) equipped with an auto-injector, high-pressure gradient pump, and UV–Vis detector. A reverse-phase C18 column (ZORBAX Eclipse Plus C18, 4.6 × 150 mm, pore size 5 μm, Agilent Tech., CA) was used for separation. The mobile phase consisting of an isocratic system using acetonitrile:DW (55:45) solvent was delivered at a flow rate of 1 ml/min using a pump. The column effluent was detected at 230 nm, and the concentration of DTX was calculated based on a linear calibration curve of standard DTX. The drug loading content and efficiency were calculated by the following equations:

Drug loading content (%)=(Weight of loaded drug in the micelles)/(Weight of total drug-loaded micelles)×100

Drug loading efficiency (%)=(Weight of loaded drug in the micelles)/(Weight of drug initially added to formulations)×100

#### Particle size measurement

The particle sizes (effective hydrodynamic diameters) of micelles were measured by photon correlation spectroscopy using Zetasizer Nano-ZS (Malvern Instruments, UK) equipped with the Multi Angle Sizing Option (BI-MAS). Measurements were performed in a thermostatic cell at a scattering angle of 173° (backscatter, NIBS Default). Software provided by the manufacturer was used to calculate effective hydrodynamic diameter values. The average *D*_eff._ was calculated from three measurements of each sample (*n* = 3).

#### Morphology

Diluted DTBM dispersions in DW (0.1 mg/ml) were deposited onto a slide glass and dried in a vacuum. The morphology of DTBM was observed after platinum (Pt) coating using field emission scanning electron microscopy (FE-SEM, Sigma, Carl Zeiss, Germany).

#### Redispersion of the micellar formulation

To prepare a powder state of docetaxel-loaded triblock micelles (DTBM-R), the effect of various surfactants (PVA, Pluronic F68, PEG 5000 (PEG 5K), PEG 2000 (PEG 2K), PEG 400, and tween 80) and protectants (trehalose, sucrose, lactose, glycine, and D-mannitol (Man)) on redispersion of polymeric micelle without docetaxel (blank micelle) was studied.

Blank micelle (0.1%) was prepared by dialysis method using DW and PBS and collected. Surfactants and protectants were added to blank micelle solution (9 ml) before lyophilization and it was lyophilized in a freeze dryer (FDB-5503, Operon, Gimpo-si, Gyeonggi-do, Korea). The powder state of the formulation was reconstituted by simply adding 9 ml of DW with gentle manual agitation in 10-ml vials (clear 10-ml crimp finish vials, with thicker sealing lip, SUPELCO, Bellefonte, PA) (Oh et al., 2008). The rehydration process was observed visually.

Based on the optimization using blank micelle, DTBM-R was prepared with DTBM, PEG 2K (0.5 w/v %), and Man (0.5 w/v %) using the procedure described above. Briefly, DTBM, PEG 2000, and Man were mixed homogenously in 10 ml glass vials before lyophilization. DTBM was reconstituted by gentle agitation after addition of 9 ml of DW. Particle size and DTX recovery of the formulations were compared using DLS and HPLC, respectively.

#### Drug release profile

To determine the drug release, DTBM-R and the commercial product (Nanoxel M) with 25 mg of DTX were dispersed in PBS at pH 7.4 and transferred to a Spectra/Por dialysis membrane tube with a molecular weight cutoff of 3500. Each membrane tube was immersed in a vial containing 25 ml of 0.9% NaCl solution. The release of DTX from DTBM-R was tested under mechanical shaking (100 rpm) at 37 °C. At predetermined time intervals, 1 ml samples of the outer phase of the dialysis membrane were collected for analysis of drug concentration and replaced with the same amount of fresh medium to maintain a sink condition (Sandhu et al., 2015). The released DTX in the continuous outer phase was evaluated by HPLC analysis.

#### In vitro anticancer effect

KB cells were maintained in RPMI 1640 medium supplemented with 10% fetal bovine serum and grown in a humidified incubator at 37 °C in a 5% CO_2_ atmosphere. Cells were harvested from growing monolayers and seeded into 96-well plates (5000 cells in 100 μl of RPMI 1640 per well) for 24 h prior to cytotoxicity tests. After the incubation, the media was removed, and cells were washed with DPBS. Cells were treated with the Nanoxel M, DTBM, or DTBM-R at different concentrations by incubation at 37 °C in 5% CO_2_ for 48 h. The viability of KB cells was determined by CCK assay. Briefly, fresh medium containing CCK solution (10 vol%) was added to each well and the plate was incubated for an additional 2 h. The absorbance of each well was then read on a Flexstation 3 microplate reader (Molecular Devices, Sunnyvale, CA) using a wavelength of 450 nm. The viability of cells treated with samples was compared with non-treated cells in the same medium. IC_50_ values were calculated with GraphPad Prism 5 software. (GraphPad Software, La Jolla, CA, USA).

#### Animal care

Animal care and all animal experiments were performed in accordance with the National Institute of Health Guidelines Principles of Laboratory Animal Care and the Animal Protection Law in Republic of Korea and were approved by the Institutional Animal Care and Use Committee (IACUC) of Chung-Ang University, Seoul, Republic of Korea. Tumor xenografts were established by subcutaneous injection of 1 × 10^7^ KB cells suspended in 0.1 ml of DPBS into BALB/c nude mice (Orient Bio Inc., Seoul, Korea). Tumor volume was calculated with the following equation: tumor volume = length × (width)^2^/2 (Duncan, 2003; Oh et al., 2013, 2014; Kwag et al., 2014). Studies of biodistribution and anticancer effects of the micelles were started when the tumor volume reached approximately 10–30 mm^3^.

#### Biodistribution of drug-loaded micelles

For near-infrared fluorescence real-time tumor imaging, polymeric micelles containing 10 wt% of Cy5.5–PEG–PLA–PEG and 90 wt% of PEG–PLA–PEG, which has no functional groups, in 0.9 M NaCl were injected into the tail vein of mice bearing KB tumors. The biodistribution of micelles at different time points after injection was monitored using a Fluorescence *In Vivo* Imaging System (FOBI system, Neo Science, Suwon, Korea) with a red channel for Cy5.5. At 24 h post-injection, the tumor and other main organs were isolated to check for accumulation of micelles. The *in vivo* and *ex vivo* fluorescence levels were determined with NEOimage software (Neo Science, Suwon, Korea).

#### In vivo anticancer efficacy and toxicity

BALB/c nude mice bearing tumors were randomly divided into four groups. PEG–PLA–PEG, Nanoxel M, and DTBM-R were injected intravenously into tumor-bearing mice through tail veins at a dose of 2 mg/kg. Mice in the control group received intravenous injection of saline (0.2 ml) into the tail vein. The relative tumor volume (%) was defined as the volume percentage of a tumor at predetermined time intervals (0–15 days) relative to the initial volume of the tumor. Changes in tumor sizes and body weights of mice were monitored every 3 days for 15 days.

## Results and discussion

### Optimization of the micellar formulation

Considering ‘particle isolation’ hypothesis (Allison et al., 2000) and ‘water replacement hypothesis (Crowe et al., 1994; Allison et al., 1998; Chen et al., 2010), the protection level against lyophilization process would be depend on properties and concentrations of the excipients and nanoparticle (Picco et al., 2018). To determine the best combination of surfactant and protectants for DTBM-R, the effect of various excipients on micelle reconstitution were checked using polymeric micelle without DTX (blank micelle) by DLS.

**Figure 1.**
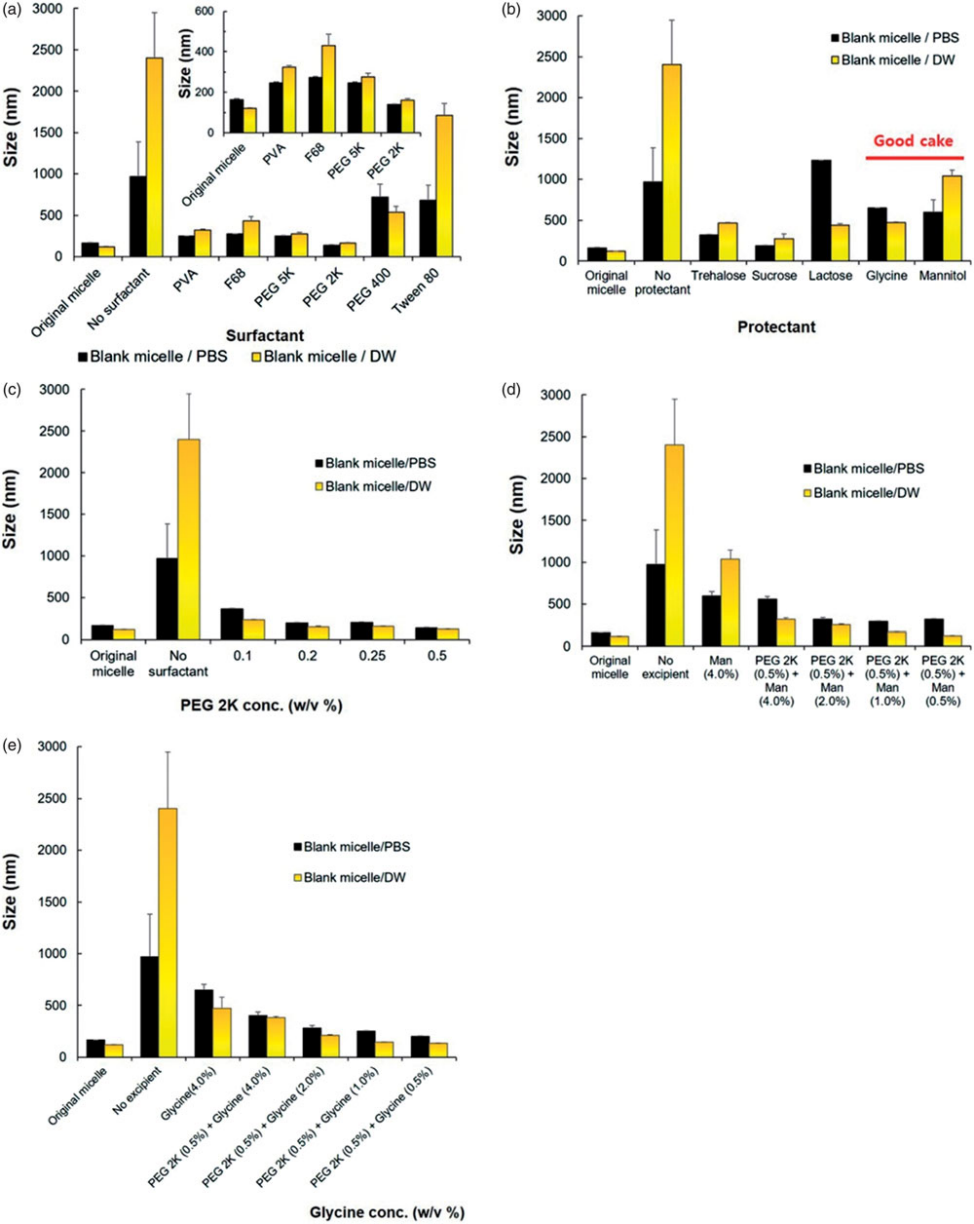
Optimization of the formulation by checking the particle size of reconstituted blank micelles using DLS: effect of various (a) surfactants (0.5%) and (b) protectants (4%) on the reconstitution of blank micelles (0.1%) made by PBS (blank micelle/PBS) and DW (blank micelle/DW). (c) Particle size of the reconstituted blank micelles depending on the amount of PEG 2K. Effect of (d) Man and (e) glycine on the particle size of reconstituted blank micelle. Combination of surfactant (PEG 2K, 0.5%) and protectant (Man or glycine, 0.5%) showed the most similar particle size of reconstituted micelle with the original one without any excipients before lyophilization.

Blank micelles prepared by PBS and DW showed approximately 163 nm and 120 nm, respectively. First, various surfactants (0.5 w/v%) were added and lyophilized to check the effect on micelle reconstitution using 0.1% of blank micelle (Figure 1) (Abdelwahed et al., 2006; Oh et al., 2008; Fonte et al., 2016). Reconstituted micelles using PEG 2K showed the most similar particle size to the original micelles among numerous surfactants (Figure 1(a)). Similarly, various protectants were also applied to blank micelles with relatively high amount (4 w/v%). Even though other protectants showed smaller particle size, glycine and Man were selected as protectants with high potential to enable successful redispersion. Lyophilized micelles including glycine and Man showed proper appearance with ‘good cake’ without any collapsed state that could prevent successful reconstitution of the formulation (Figure 1(b)) (Fonte et al., 2016).

To decrease the content of PEG 2K, particle size of blank micelle after redispersion was compared depending on the concentration of PEG 2K. It was optimized as 0.5 w/v % because reconstituted micelles with a lower concentration of PEG 2K showed a larger particle size (Figure 1(c)). To determine the protectant, various combination of protectants with PEG 2K (0.5 w/v%) was added to the blank micelles. Since reconstituted micelle prepared using Man (0.5 w/v%) showed the smallest particle size with approximately 125 nm, Man was selected as protectant for the micellar formulation (Figure 1(d,e)).

Furthermore, micellar formulation prepared by DW showed smaller particle size than one prepared by PBS. The presence of salt would affect the particle size of the formulation by dehydration of PEG that could result in a modification of inter- and intra-micellar interactions (Carale et al., 1994; Jain et al., 1999; Mata et al., 2005). Considering the tumor targeting ability, DTBM and DTBM-R was prepared by DW to form nanoparticles less than 200 nm (Maeda et al., 2000; Fang et al., 2011).

### Characterization of DTBM

**Figure 2.**
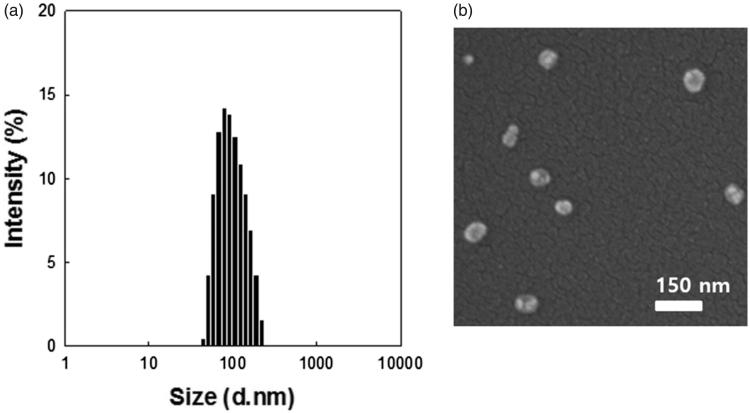
Characterization of DTBM (targeting 10%). (a) Particle size distribution by DLS and (b) morphologies by FE-SEM.

DTX, a common anticancer drug, was chosen as a typical insoluble drug for loading into micelles based on PEG–PLA–PEG triblock copolymers using the dialysis method. DTBMs prepared for 10, 20, and 30 wt% target drug loading amounts are characterized in Table 1. As the target loading amount (wt%) of DTX increased, the DTBMs showed slightly decreased particle size, the polydispersity index (PDI), and drug loading efficiency. When the target loading amount was increased to 20 and 30 wt%, the DTX loading content in DTBM decreased compared with a 10 wt% targeted loading amount. Based on these results, the loading capacity of DTBM for DTX is shown to be approximately 7.4 wt%. High DTX loading capacity in the micelles may be due to the hydrophobic PLA block that enabled the physical entrapment of hydrophobic drug in polymeric micelles by hydrophobic interaction between the drug molecule and the hydrophobic core of micelles (Shuai et al., 2004; Gao et al., 2012; Zhang et al., 2014; Hoang et al., 2017a; Qi et al., 2017). DTBM prepared with 10% DTX loading exhibited particles of 125 nm in size with a spherical structure and narrow size distribution in DW (Figure 2).

**Table 1.** Characterization of DTBM (*n* = 3).

Targetcontent (%)^a^	Loadingcontent (%)^b^	Loadingefficiency (%)^c^	Size (nm)	PDI
10	7.4	81.9	125 ± 2.7	0.24 ± 0.01
20	10.9	65.3	84 ± 2.0	0.26 ± 0.02
30	12.4	53.5	83 ± 4.2	0.29 ± 0.02

^a^ Target content (%)=(weight of targeted drug amount in micelles)/(weight of block copolymers in formulation)×100.

^b^ Drug-loading content (%)=(weight of loaded drug in micelles)/(weight of total drug loaded micelle)×100.

^c^ Drug-loading efficiency (%)=(weight of loaded drug in micelles)/(weight of drug initially added to formulations)×100. PDI: polydispersity index determined by dynamic light scattering.

### Optimization of DTBM-R

**Figure 3.**
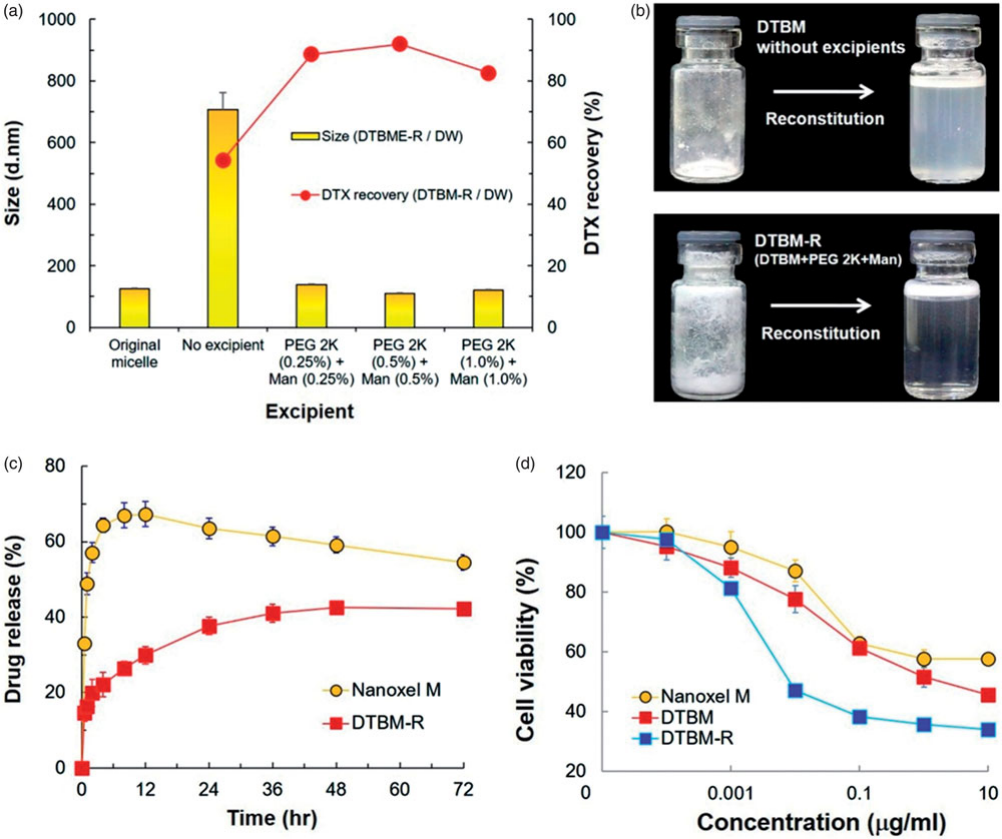
Optimization and characterization of DTBM-R. (a) Effect of excipients with different concentrations on reconstitution of DTBM-R using PBS and DW that includes PEG 2K (0.5 w/v %) as a surfactant and Man (0.5 w/v %) as a protectant. (b) Comparison of reconstitution: Successful reconstitution of DTBM-R containing PEG and Man and poor reconstitution of lyophilized polymeric micelles without excipients. (c) Drug release profile and (d) cytotoxicity in KB cells treated with the Nanoxel M, DTBM, or DTBM-R for 48 h.

Since PEG 2K and Man were determined as the best excipients for the micellar formulation (Figure 1), they were further optimized for the formulation of DTBM with successful reconstitution (DTBM-R). The effect of the excipients concentration was compared by particle size of the reconstituted micelles and DTX concentration recovery (Figure 3(a)). DTBM-R containing PEG 2K (0.5 w/v%) and Man (0.5 w/v%) showed the nano-sized particles of approximately 110 nm and the highest DTX recovery greater than 90%. Successful reconstitution of DTBM-R was also visualized by optical imaging compared with poor reconstitution of DTBM without PEG 2K and Man (Figure 3(b)). PEG 2K and Man stabilized nanoparticles and prevented their aggregation during lyophilization by acting as a surfactant and a protectant against freezing and drying stresses (Abdelwahed et al., 2006; Fonte et al., 2016). Because of the presence of PEG on the surface of micelles, the proximity among the micellar particles, and addition of PEG 2K as a surfactant, intra- and inter particular connections of crystallized PEG might be formed during lyophilization, which could prohibit the reconstitution of the micelles (Abdelwahed et al., 2006). The addition of Man would maintain the PEG corona in a pseudo-hydrated state through intramolecular hydrogen-bonding that enables the redispersion of micellar formulation (De Jaeghere et al., 1999; Zambaux et al., 1999).

### Drug release and cell viability

To investigate DTX release from the formulation, Nanoxel M and DTBM-R were exposed to PBS at pH 7.4 (Figure 3(c)). Nanoxel M showed a drastic increase in DTX release. It gave a maximum release rate of drug at 12 h followed by slight decrease. At 12 h, the drug released from the commercial product might be entirely dissolved as a supersaturated form in the release medium. Moreover, with the elapsed time, the slight decrease after the maximum at 12 h would be caused by recrystallization or precipitation of the solubilized drug released from micelles (Xie et al., 2017; Schver & Lee, 2018). In contrast, DTBM-R showed sustained drug release with less than 50% maximum cumulative drug release over 72 h. The sustained release of DTX from DTBM-R would minimize drug loss and reduce systemic toxicity during blood circulation (Qureshi et al., 2017; Su et al., 2018). This might be attributed to the high stability of DTBM-R caused by PEG–PLA–PEG (2K–6K–2K). As previously reported, the triblock copolymer formed stable polymeric micelles and sustained a particle size less than 200 nm for 7 days because the density and thickness of PEG on the surface of the micelle could provide high steric stabilization (Hoang et al., 2017a). Thus, the high stability of the polymeric micelle would prevent burst release of DTX and showed a potential to minimize drug loss and systemic toxicity.

DTBM-R clearly showed higher cytotoxicity than Nanoxel M in the range of concentration of DTX (Figure 3(d)). Compared to Nanoxel M (IC_50_ = 8.872 μg/ml), DTBM and DTBM-R showed approximately 28-fold and 129-fold higher efficacy against KB cells (IC_50_ = 1.924 μg/ml and 0.06874 μg/ml, respectively). Since the triblock copolymer and the other excipients do not appear to induce toxicity (Figure S2), the high toxicity of DTBM and DTBM-R might be attributed to increased translocation due to the nano-sized particles (Peer et al., 2007; van Vlerken et al., 2007; Hillaireau & Couvreur, 2009).

### Biodistribution and pharmacodynamic studies

**Figure 4.**
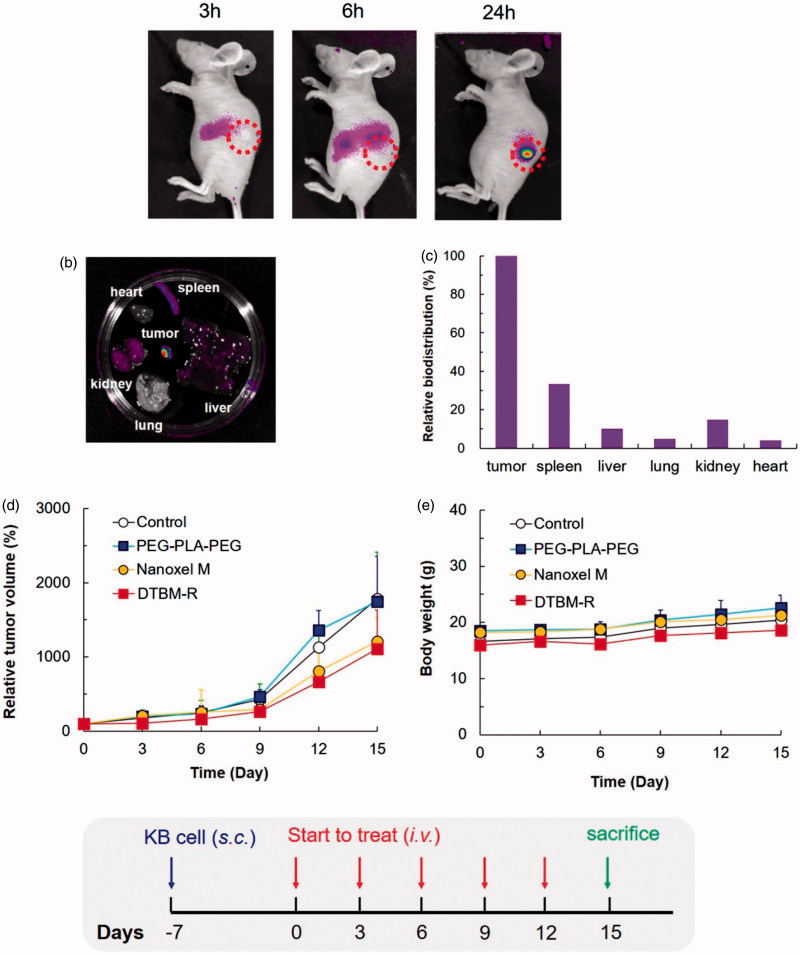
Non-invasive *in vivo* fluorescent imaging of Cy5.5-tagged micelles after intravenous injection into the tail vein of KB tumor-bearing nude mice. (a) Whole body imaging at predetermined time points after i.v. injection. (b) *Ex vivo* optical and fluorescent imaging of tumor and organs obtained 24 h post-injection. (c) Relative biodistribution of PEG-PLA-PEG micelles by quantitative fluorescence intensity (FI) of tumors and main organs. The relative biodistribution of the spleen, liver, and kidney compared to the tumor was 33.3%, 10.1%, and 14.8%, respectively. *In vivo* (d) relative tumor volume and (e) body weight in KB tumor-bearing mice injected with saline (control), PEG–PLA–PEG, Nanoxel M, docetaxel-loaded triblock micelle (DTBM) (equivalent to 2 mg/kg DTX, *n* = 3). Relative tumor volume was defined as volume ratio of tumor at predetermined time intervals (0–15 days) compared to the initial volume.

Evaluation of the tumor targeting ability of PEG–PLA–PEG in tumor-bearing nude mice by high-resolution fluorescent imaging using Cy5.5-tagged PEG–PLA–PEG revealed that the micelle gradually accumulated at tumor sites over 24 h (Figure 4(a)). At 24 h, marked concentration at tumor sites was evident. To identify the biodistribution of micelles, the nude mice were sacrificed after 24 h and excised organs were examined *ex vivo*. Representative fluorescence images and fluorescence intensity of various organs indicate that most of the micelles were intensively concentrated at tumor sites (Figure 4(b)). Relative biodistribution of PEG–PLA–PEG micelles was determined by quantitative fluorescence intensity (FI) of tumor and main organs. As expected, accumulation of the micelles at the tumor site was significantly higher than accumulation in other organs; the relative biodistribution of the spleen, liver, and kidney compared to the tumor was 33.3%, 10.1%, and 14.8%, respectively (Figure 4(c)).

Tumor growth inhibition was studied using a tumor-bearing mouse model with the same cell line (Figure 4(d)). For the *in vivo* study, the commercial product and DTBM-R were utilized with doses equivalent to 2 mg/kg DTX. Compared with the commercial product, DTBM-R showed higher tumor growth inhibition due to its tumor targeting ability. Interestingly, DTBM-R and the commercial product showed similar suppression of tumor growth. Furthermore, changes in the body weight of nude mice treated with saline, PEG–PLA–PEG (2K–6K–2K), Nanoxel M, and DTBM-R were negligible. Since the micelles would highly accumulate in the tumor, DTBM-R showed no obvious toxicity throughout the body (Figure 4(e)).

**Figure 5.**
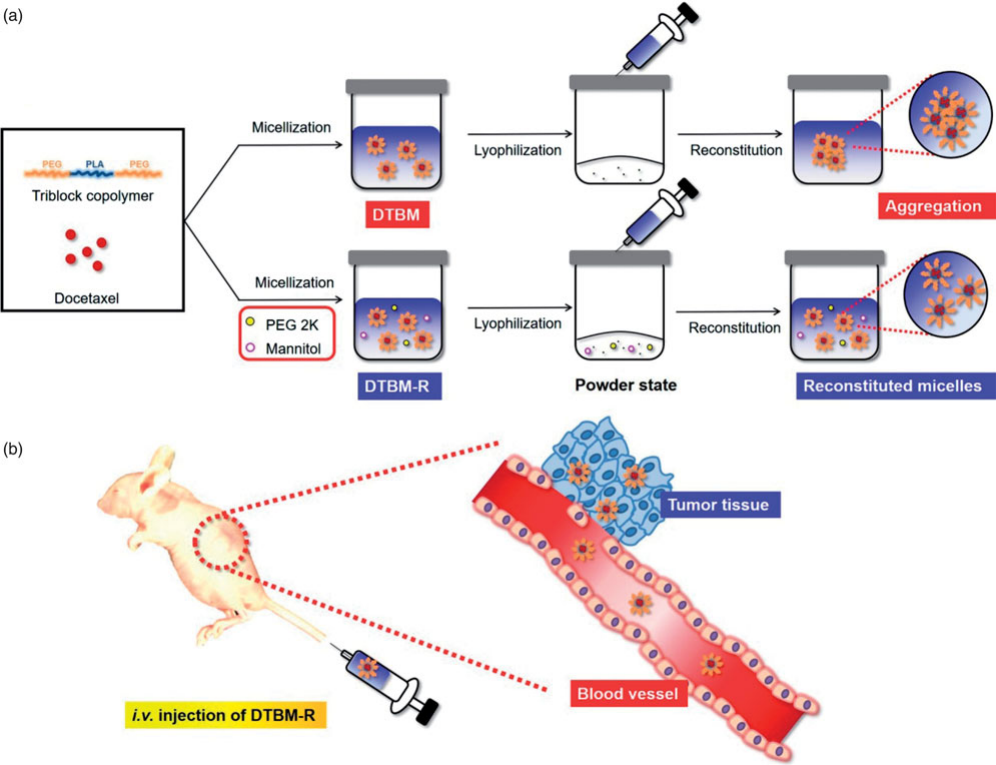
Schematic concept of docetaxel-loaded micellar formulation using PEG–PLA–PEG. (a) Preparation of DTBM-R using PEG 2K and Man for successful reconstitution and (b) proposed *in vivo* performance of DTBM-R after i.v. administration.

Based on these results, the pharmaceutical application and tumor growth inhibition by DTBM-R are summarized in Figure 5. DTBM-R could be reconstituted after lyophilization because the use of PEG 2K and Man as surfactant and protectant respectively stabilized the micelles against freezing and drying stresses and prevented their aggregation during lyophilization. DTBM-R could incorporate DTX into the hydrophobic cores of the micelles formed by hydrophobic PLA blocks. Intravenously injected DTBM-R could circulate within blood vessels without significant extravasation for a longer duration and minimized DTX loss through sustained release due to its highly stable and robust micellar structure (Song et al., 2016; Hoang et al., 2017a). The circulated DTBM-R could then be distributed and accumulate in tumor sites through the enhanced permeability and retention (EPR) effect (Maeda, 2001; Fang et al., 2011; Bertrand et al., 2014; Danhier, 2016; Qureshi et al., 2017). At the tumor site, the DTBM-Rs could release DTX and inhibit the tumor with low systemic toxicity.

## Conclusion

From the pharmaceutical development point of view, DTBM-R represents a practical formulation of anticancer nanomedicine for storage that could be reconstituted after lyophilization. The robustly formed micelles minimize systemic toxicity due to the sustained drug release. DTBM-R might also maximize antitumor efficacy through increased accumulation and release of DTX. DTBM-R with successful reconstitution could be considered as a potent nanomedicine for tumor treatment.

## Supplementary Material

Supplemental Material

Supplemental Material
