# Supplementary material for: Development of a docetaxel micellar formulation using poly(ethylene glycol)–polylactide–poly(ethylene glycol) (PEG–PLA–PEG) with successful reconstitution for tumor targeted drug delivery
Source: Drug Deliv. 2018 Jun 5;25(1):1362–71. doi: 10.1080/10717544.2018.1477865 (PMC6060706; doi:10.1080/10717544.2018.1477865)

**Development of a docetaxel micellar formulation using poly(ethylene glycol)-polylactide-poly(ethylene glycol) (PEG-PLA-PEG) with successful reconstitution for tumor targeted drug delivery**

Taehoon Sim^a^, Jae Eun Kim ^a^, Ngoc Ha Hoang ^a+^, Jin Kook Kang ^a^, Chaemin Lim ^a^, Dong Shik Kim ^b^, Eun Seong Lee ^c^, Yu Seok Youn^d^, Han-Gon Choi^b^, Hyo-Kyung Han ^e^, Kwon-Yeon Weon ^f^, and Kyung Taek Oh^a^***

*^a^ College of Pharmacy, Chung-Ang University, 84 Heukseok-ro, Dongjak-gu, Seoul 06974, Korea;*

*^b^ College of Pharmacy & Institute of Pharmaceutical Science and Technology, Hanyang University, 55 Hanyangdaehak-ro, Sangnok-gu, Ansan 426-791, Republic of Korea;*

*^c^ Department of Biotechnology, The Catholic University of Korea, 43-1 Yeokgok 2-dong, Wonmi-gu, Bucheon, Gyeonggi-do, 14662 Korea;*

*^d^ School of Pharmacy, SungKyunKwan University, 300 Cheoncheon-dong, Jangan-gu, Suwon City, 16419 Korea;*

*^e^ College of Pharmacy, Dongguk University-Seoul, Goyang, Gyeonggi-do, Korea;*

*^f^ College of Pharmacy, Catholic University of Daegu, 13-13 Hayang-ro, Hayang-eup, Gyeongsan-si, Gyeongbuk 38430, Korea*

*Correspondence to Kyung Taek Oh

Address: College of Pharmacy, Chung-Ang University, 84 Heukseok-ro, Dongjak-gu, Seoul 06974, Korea

Email: [kyungoh@cau.ac.kr](mailto:kyungoh@cau.ac.kr)

Fax: 82-2-393-3035 / Tel: 82-2-2228-7400

^+^Current affiliation: Dr. Hoang are currently working at Department of Pharmaceutics, Ha Noi University of Pharmacy, 13-15 Le Thanh Tong, Ha Noi, Viet Nam.

**Materials and Methods**

**Materials**

Methoxy poly(ethylene glycol) (mPEG, MW 2K), L-lactide ((3S)-cis-3,6-dimethyl-1,4-dioxane-2,5-dione), N,N-dicyclohexylcarbodiimide (DCC), stannous octoate (Tin(II)-2-ethylhexanoate, Sn(Oct)2), 4-dimethylaminopyridine (DMAP), succinic anhydride, pyridine, triethylamine (TEA), diethyl ether, pyrene, CDCl_3_, dimethylsulfoxide (DMSO) and dimethylformamide (DMF) were purchased from Sigma-Aldrich (St. Louis, MO, USA). Diethyl ether and hexane were purchased from Samchun Chemical (Gangnam-gu, Seoul, Korea). Tetrahydrofuran (THF), toluene, acetone, and dichloromethane (DCM) were purchased from Honeywell Burdick & Jackson® (Muskegon, MI, USA). Polystyrene standards were obtained from Scientific Polymer Products Inc. (Ontario, NY, USA). Cyanine 5.5 amine (Cy.5.5 amine) was purchased from Lumiprobe (Waltham, MA, USA). KB cells were obtained from Korean Cell Line Bank (Jongno-gu, Seoul, Korea). RPMI 1640 medium, DPBS, penicillin-streptomycin solution, trypsin-EDTA solution, and fetal bovine serum (FBS) were purchased from Welgene (Gyeongsan-si, Gyeongsangbuk-do, Korea). Cell Counting Kit-8 (CCK-8) was purchased from Dojindo Molecular Technologies, Inc. (Rockville, MD, USA). PEG-PLA-PEG triblock copolymers were synthesized using procedures described previously (Hoang *et al.*, 2016, Song *et al.*, 2016, Hoang *et al.*, 2017b). Poly(ethylene glycol) 2000 (MW 2kDa, PEG 2K), D-Mannitol (Man) were purchased from Sigma-Aldrich (St. Louis, MO, USA).

**Methods**

*Synthesis of PEG-PLA-PEG*

First, PEG-PLA diblock copolymers were synthesized by ring-opening polymerization of L-lactide in the presence of PEG as initiator at 120°C for 24 h, using Sn(Oct)_2_ as catalyst and toluene as a solvent. PEG-PLA diblock copolymers synthesized by this method were precipitated by addition of the reaction mixtures to an excess amount of cold diethyl ether. The precipitates were then filtered and dried in vacuum for 2 days.

Carboxylated PEG-PLA (PEG-PLA-COOH) was prepared by the reaction of PEG-PLA with succinic anhydride in the presence of DMAP, TEA, and pyridine. First, PEG-PLA, succinic anhydride, and DMAP were reacted in 20 mL of DCM for 3 h, followed by the addition of TEA and pyridine. The reaction was carried out overnight and PEG-PLA-COOH was obtained by precipitation in an excess amount of diethyl ether. The final product was filtrated and dried in vacuum for 2 days.

To prepare PEG-PLA-PEG triblock copolymer, the synthesized PEG-PLA-COOH and PEG were conjugated by Steglich esterification in DCM using DCC and DMAP as coupling reagent and catalyst, respectively. The reaction was carried out overnight and the final products were obtained by precipitation in an excess amount of diethyl ether, subsequent filtration, and drying in vacuum for 2 days (**Scheme S1a**).

For near-infrared fluorescence real-time tumor imaging, cy 5.5 amine (1 mol ratio) was reacted with monocarboxylated PEG-PLA-PEG using NHS chemistry in DMSO for 12 h. Free Cy5.5 amine in the reactant mixture was removed by dialysis against 1 L of distilled water for 24 h. Cy5.5-PEG-PLA-PEG powder was obtained by lyophilization (**Scheme S1b**).

*Characterization of PEG-PLA-PEG*

^1^H-NMR spectroscopy and gel permeation chromatography (GPC) were used to determine the MW and composition of the block copolymers at each step. (Hoang *et al.*, 2017a). ^1^H-NMR was performed using NMR 600 MHz instrument (Delta, JEOL, MA, USA). CDCl_3_ was used as a solvent for the analysis of block copolymers. The MW of the PLA segment was determined from the ^1^H-NMR spectrum by examining the peak intensity ratio of the methylene proton of the PLA segment (COCH(CH_3_)O: δ = 5.2 ppm) and the methylene protons of the PEG segment (OCH_2_CH_2_: δ = 3.6 ppm) based on the number-average MW of PEG (Yasugi *et al.*, 1999). Number- and weight-average MWs (M_n_ and M_w_, respectively) as well as polydispersity index (M_w_/M_n_, PDI) of the copolymers were determined by GPC (Agilent 1200 series, Agilent Tech., CA, USA) equipped with a refractive index detector. THF was used as the mobile phase at a flow rate of 1.0 mL/min. Column temperature was set at 30°C. The copolymers were dissolved in THF, filtered, and injected into a PLgel 10 mm MIXED-B column (Agilent Tech., CA, USA). The M_w_ values of block copolymer were calculated based on the calibration curve generated from a series of polystyrene standards (Scientific Polymer Products Inc., Ontario, NY) (Jain *et al.*, 2010).

*CMC determination*

Fluorescence measurement for critical micelle concentration (CMC) was performed using a Scinco FS-2 fluorescence Spectrometer (Gangnam-gu, Seoul, Korea) equipped with polarizers for excitation and emission of light beams. Pyrene was used as the fluorescent probe. The sample solutions were prepared by addition of pyrene solution in acetone to empty amber vials. After evaporation, micelle solutions with different concentrations of triblock copolymers were added to the vials, resulting in a final pyrene concentration of 6 × 10^−7^ M. These samples were stirred overnight at room temperature. Excitation spectra of pyrene in samples were recorded at *λ_em_*=374 nm at room temperature. CMC was estimated by plotting the ratio of *I*_336_ (fluorescent intensity at 336 nm) to *I*_334_ (fluorescent intensity at 334 nm) of the excitation spectra against the logarithms of the copolymer concentrations. CMC was defined as the crossover point of low copolymer concentrations on this plot (Lysenko *et al.*, 2002, Han *et al.*, 2003).

*Toxicity of PEG-PLA-PEG and other excipients*

KB cells were maintained in RPMI 1640 medium supplemented with 10% fetal bovine serum and grown in a humidified incubator at 37°C and a 5% CO_2_ atmosphere. KB cells harvested from growing monolayers were seeded into 96-well plates (5000 cells in 100 μL of RPMI 1640 per well) for 24 h prior to cytotoxicity tests. After incubation, the media was removed, and cells were washed with DPBS. Cells were treated with different concentrations of PEG-PLA-PEG or PEG-PLA-PEG with PEG (0.5 w/v %) and Man (0.5 w/v %) and incubated at 37°C in 5% CO_2_ for 48 h. The viability of KB cells was determined by CCK assay. Briefly, fresh medium containing CCK solution (10 vol%) was added to each well and the plate was incubated for an additional 2 h. The absorbance of each well was read on a Flexstation 3 microplate reader (Molecular Devices, Sunnyvale, CA, USA) using a wavelength of 450 nm. The viability of treated cells was compared with that of non-treated cells in the same medium. IC_50_ values of the samples were calculated with GraphPad Prism 5 software.

**Results and discussion**

*Characterization of PEG-PLA-PEG*

PEG-PLA-PEG triblock copolymers targeting molecular weight of 2K-6K-2K were synthesized by Steglich esteriﬁcation between PEG-PLA-COOH diblock copolymers and PEG (**Scheme S1**) [22]. Succinic anhydride was used as the link and the reaction was performed under mild conditions (at room temperature) that negligibly affected the PLA backbone. The molecular weight of the synthesized triblock copolymer was conﬁrmed by ^1^H NMR (**Figure S1**) and GPC (**Table S1**). The peaks at 3.3 and 3.6 ppm were assigned to proton **a** of methoxy groups and proton **b** of ethylene of PEG blocks. The peaks at 5.2 and 1.6 ppm were assigned to protons **c** and **d**, respectively, of PLA. An obvious increase on the ratios of PEG and PLA proton peak intensities was observed in triblock copolymers compared with diblock copolymers. Importantly, the MW of a block copolymer conﬁrmed by ^1^H NMR was consistent with that determined by GPC. The triblock copolymers showed low PDI, indicating narrow polymer molecular distributions resulting in the homogeneous formation of micelles.

*CMC determination*

The ﬂuorescence spectroscopy method was employed for CMC measurement using pyrene, which is highly hydrophobic and preferentially migrates into the hydrophobic core of micelles in aqueous solutions as a ﬂuorescent probe (Kalyanasundaram and Thomas, 1977). Pyrene shows weak ﬂuorescent intensity in a polar environment (e.g., aqueous solutions) but shows strong ﬂuorescent intensity in a non-polar environment, inducing a consequent sharp increase in the ratio *I_1_/I_3_* at CMC that can determine the CMC of triblock copolymers (Kalyanasundaram and Thomas, 1977, Lee *et al.*, 2003).

Polymeric micelles of PEG-PLA-PEG (2K-6K-2K) had a very low CMC value of 16.21 μg/mL (**Table S1**) indicating high thermodynamic stability. The CMC was similar to that of a previous report (approximately 12 μg/mL) (Hoang *et al.*, 2017a); therefore PEG-PLA-PEG is assumed be delivered to the tumor site without drug loss during systemic circulation after injection (Allen *et al.*, 1999, Rapoport, 2007, Wiradharma *et al.*, 2009, Ebrahim Attia *et al.*, 2011). CMC deﬁnes the thermodynamic stability of the micelles and is a key player in stabilizing micelles. Only single chains of block copolymers exist below CMC in an aqueous environment, whereas both micelles and single chains of block copolymers co-exist above it. Thus, micelles based on block copolymers with low CMC will probably have a high chance of reaching the target site without dissociating into single chains and releasing the loaded drug.

*Toxicity of PEG-PLA-PEG and other excipients*

The toxicity of PEG-PLA-PEG and other excipients was examined using CCK assay as for the *in vitro* cell viability study. As shown in **Figure S2**, PEG-PLA-PEG showed no toxicity in the range of polymer concentration of 0.0001 – 100 μg/ml. Furthermore, KB cells treated with PEG-PLA-PEG with PEG 2K (0.5 w/v %) and mannitol (0.5 w/v%) also showed good survival. There was no toxicity for the treatment with the maximum concentration of DTX used in this study at 100 μg/ml PEG-PLA-PEG with the other excipients (maximum DTX conc. = 10 μg/ml). It is considered that PEG-PLA-PEG has less potential to increase the toxicity of DTBM and DTBM-R *in vitro* and *in vivo.*

**References**

Allen, C., Maysinger, D. & Eisenberg, A., 1999. Nano-engineering block copolymer aggregates for drug delivery. *Colloids and Surfaces B: Biointerfaces,* 16**,** 3-27.

Ebrahim Attia, A.B., Ong, Z.Y., Hedrick, J.L., Lee, P.P., Ee, P.L.R., Hammond, P.T. & Yang, Y.Y., 2011. Mixed micelles self-assembled from block copolymers for drug delivery. *Current opinion in colloid & interface science,* 16**,** 182-194.

Han, S.K., Na, K. & Bae, Y.H., 2003. Sulfonamide based pH-sensitive polymeric micelles: physicochemical characteristics and pH-dependent aggregation. *Colloids and Surfaces A: Physicochemical and Engineering Aspects,* 214**,** 49-59.

Hoang, N.H., Lim, C., Sim, T., Lee, E.S., Youn, Y.S., Kim, D. & Oh, K.T., 2016. Characterization of a triblock copolymer, poly (ethylene glycol)-polylactide-poly (ethylene glycol), with different structures for anticancer drug delivery applications. *Polymer Bulletin***,** 1-15.

Hoang, N.H., Lim, C., Sim, T., Lee, E.S., Youn, Y.S., Kim, D. & Oh, K.T., 2017a. Characterization of a triblock copolymer, poly(ethylene glycol)-polylactide-poly(ethylene glycol), with different structures for anticancer drug delivery applications. *Polymer Bulletin,* 74**,** 1595-1609.

Hoang, N.H., Lim, C., Sim, T. & Oh, K.T., 2017b. Triblock copolymers for nano-sized drug delivery systems. *Journal of Pharmaceutical Investigation,* 47**,** 27-35.

Jain, A.K., Goyal, A.K., Mishra, N., Vaidya, B., Mangal, S. & Vyas, S.P., 2010. PEG–PLA–PEG block copolymeric nanoparticles for oral immunization against hepatitis B. *International journal of pharmaceutics,* 387**,** 253-262.

Kalyanasundaram, K. & Thomas, J., 1977. Environmental effects on vibronic band intensities in pyrene monomer fluorescence and their application in studies of micellar systems. *Journal of the American Chemical Society,* 99**,** 2039-2044.

Lee, E.S., Shin, H.J., Na, K. & Bae, Y.H., 2003. Poly (l-histidine)–PEG block copolymer micelles and pH-induced destabilization. *Journal of controlled release,* 90**,** 363-374.

Lysenko, E.A., Bronich, T.K., Slonkina, E.V., Eisenberg, A., Kabanov, V.A. & Kabanov, A.V., 2002. Block ionomer complexes with polystyrene core-forming block in selective solvents of various polarities. 1. Solution behavior and self-assembly in aqueous media. *Macromolecules,* 35**,** 6351-6361.

Rapoport, N., 2007. Physical stimuli-responsive polymeric micelles for anti-cancer drug delivery. *Progress in Polymer Science,* 32**,** 962-990.

Song, H.-T., Hoang, N.H., Yun, J.M., Park, Y.J., Song, E.H., Lee, E.S., Youn, Y.S. & Oh, K.T., 2016. Development of a new tri-block copolymer with a functional end and its feasibility for treatment of metastatic breast cancer. *Colloids and Surfaces B: Biointerfaces,* 144**,** 73-80.

Wiradharma, N., Zhang, Y., Venkataraman, S., Hedrick, J.L. & Yang, Y.Y., 2009. Self-assembled polymer nanostructures for delivery of anticancer therapeutics. *Nano Today,* 4**,** 302-317.

Yasugi, K., Nagasaki, Y., Kato, M. & Kataoka, K., 1999. Preparation and characterization of polymer micelles from poly (ethylene glycol)-poly (D, L-lactide) block copolymers as potential drug carrier. *Journal of controlled release,* 62**,** 89-100.

**Table S1.** Characterizations of di- and triblock copolymers (PEG-PLA and PEG-PLA-PEG)

| Targeting MW (Da) | PEG-PLA | | | PEG-PLA-PEG | | | | |
| --- | --- | --- | --- | --- | --- | --- | --- | --- |
|  | *M*_n_ | | PDI ^b^ | *M*_n_ | | PDI ^b^ | CMC  (µg/ml) | Final Structure ^c^ |
|  | ^1^H NMR | GPC ^a^ |  | ^1^H NMR | GPC ^a^ |  |  |  |
| 2K-6K-2K | 8154 | 8418 | 1.248 | 9933 | 10792 | 1.159 | 16.21 | 2K-5.9K-2K |


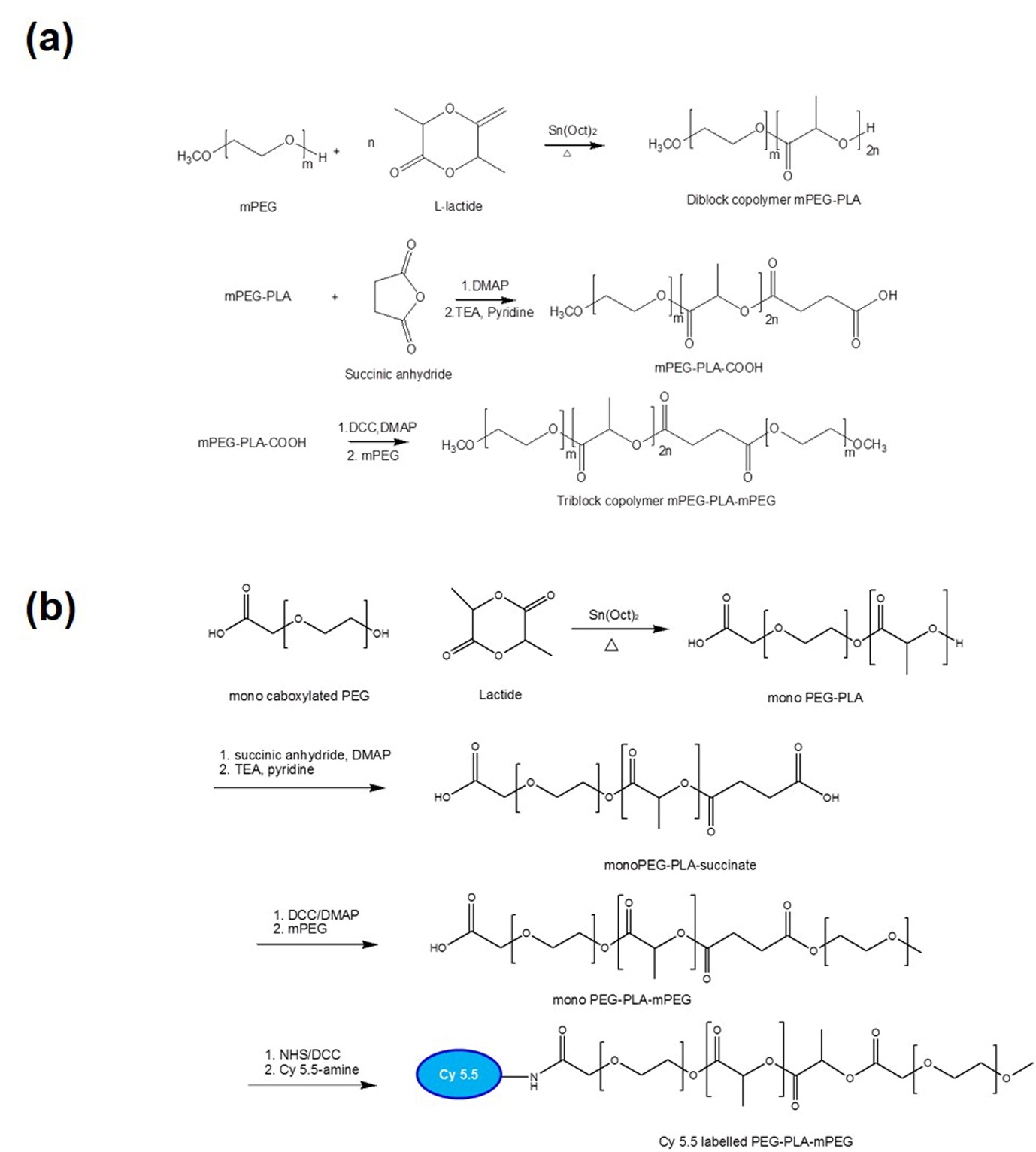


**Scheme S1.** Synthetic route for (a) mPEG-PLA-mPEG triblock copolymer and (b) Cy 5.5-tagged PEG-PLA-PEG**.**


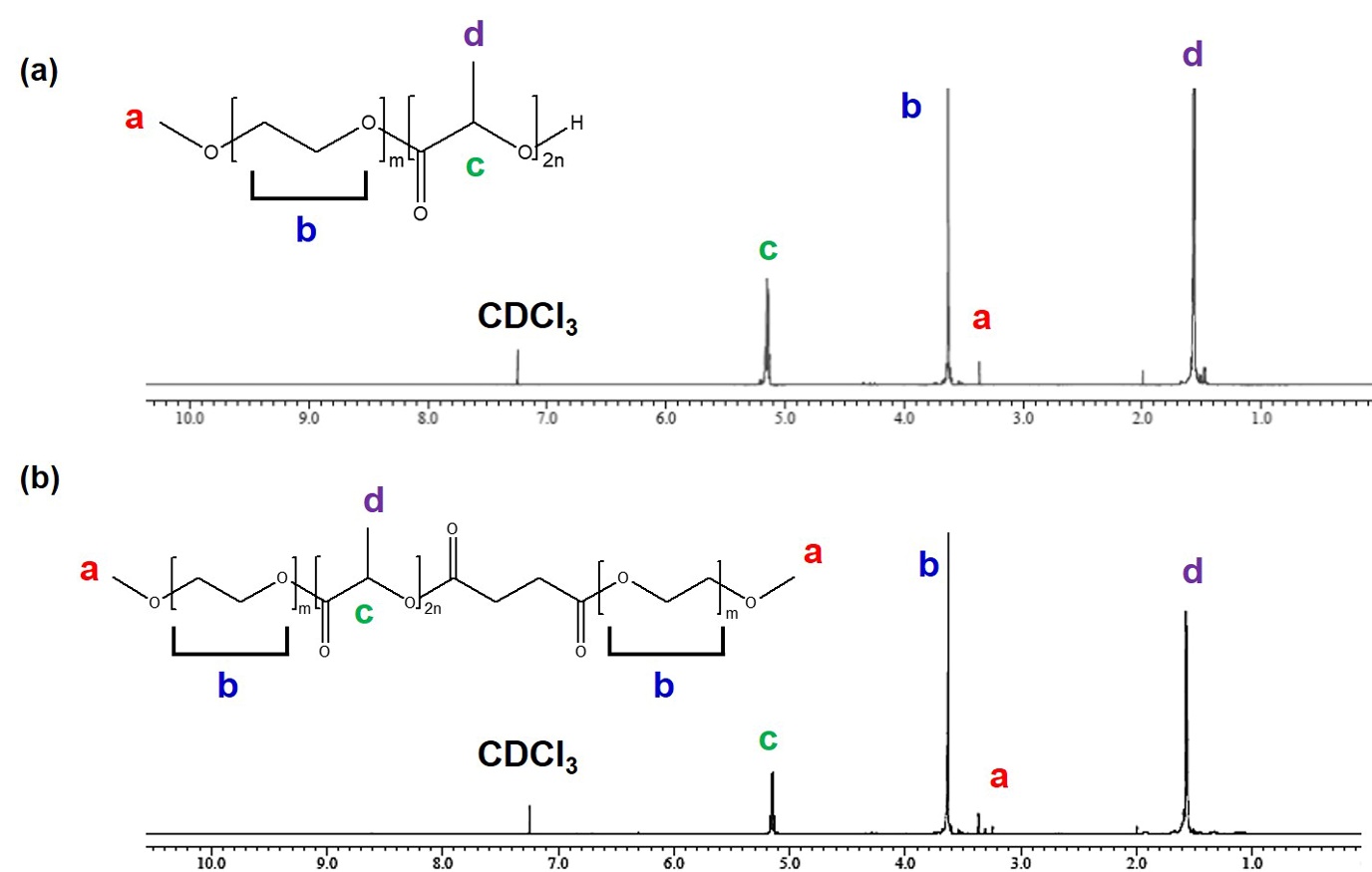


**Figure S1.** ^1^H NMR spectrum of (a) PEG-PLA (2K-6K) and (b) PEG-PLA-PEG (2K-6K-2K) using CDCl_3_ as solvent.


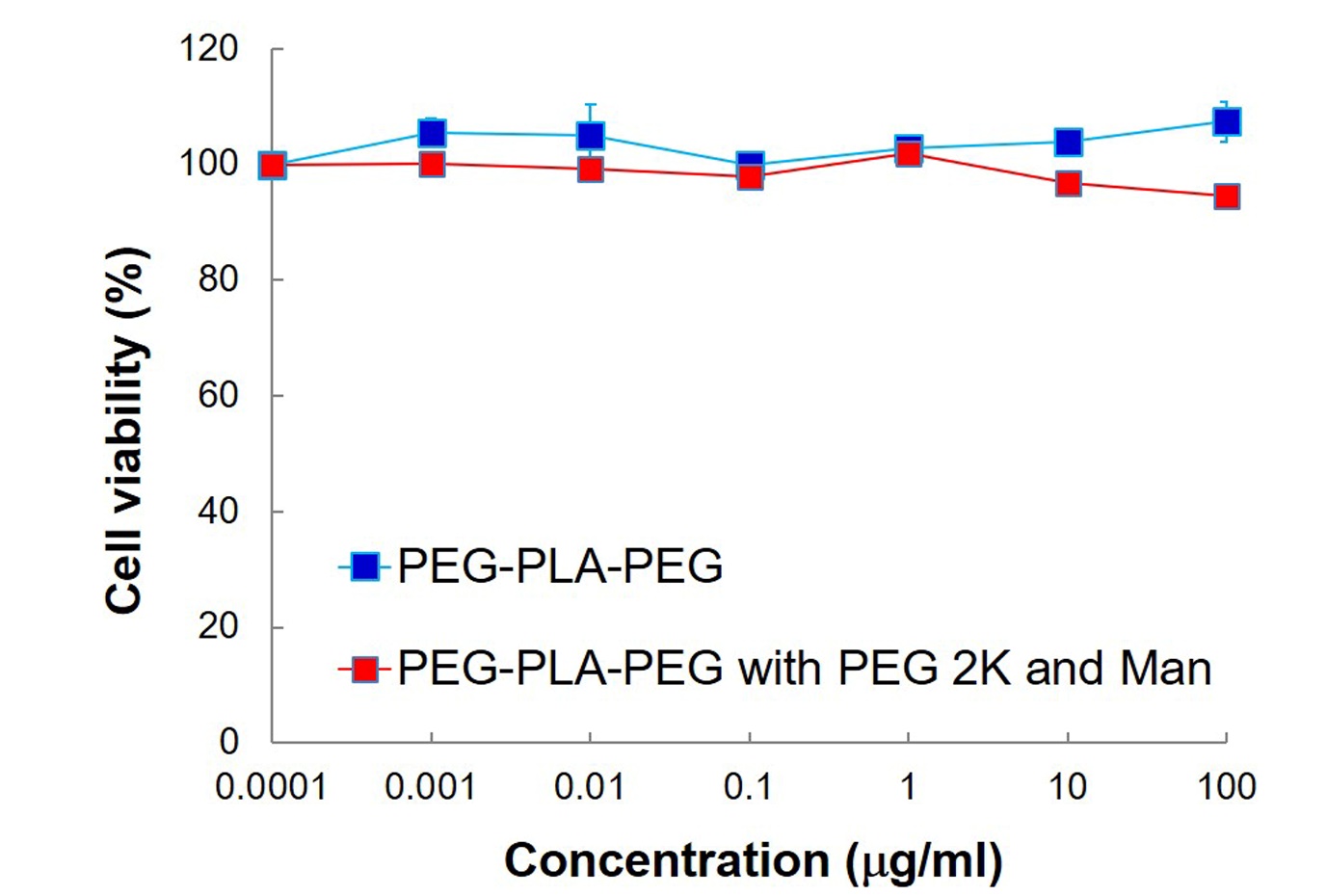


**Figure S2.** Toxicity of PEG-PLA-PEG and excipients.

**Figure captions**

**Scheme S1.** Synthetic route for (a) mPEG-PLA-mPEG triblock copolymer and (b) Cy 5.5-tagged PEG-PLA-PEG**.**

**Figure S1.** ^1^H NMR spectrum of (a) PEG-PLA (2K-6K) and (b) PEG-PLA-PEG (2K-6K-2K) using CDCl_3_ as solvent.

**Figure S2.** Toxicity of PEG-PLA-PEG and excipients.


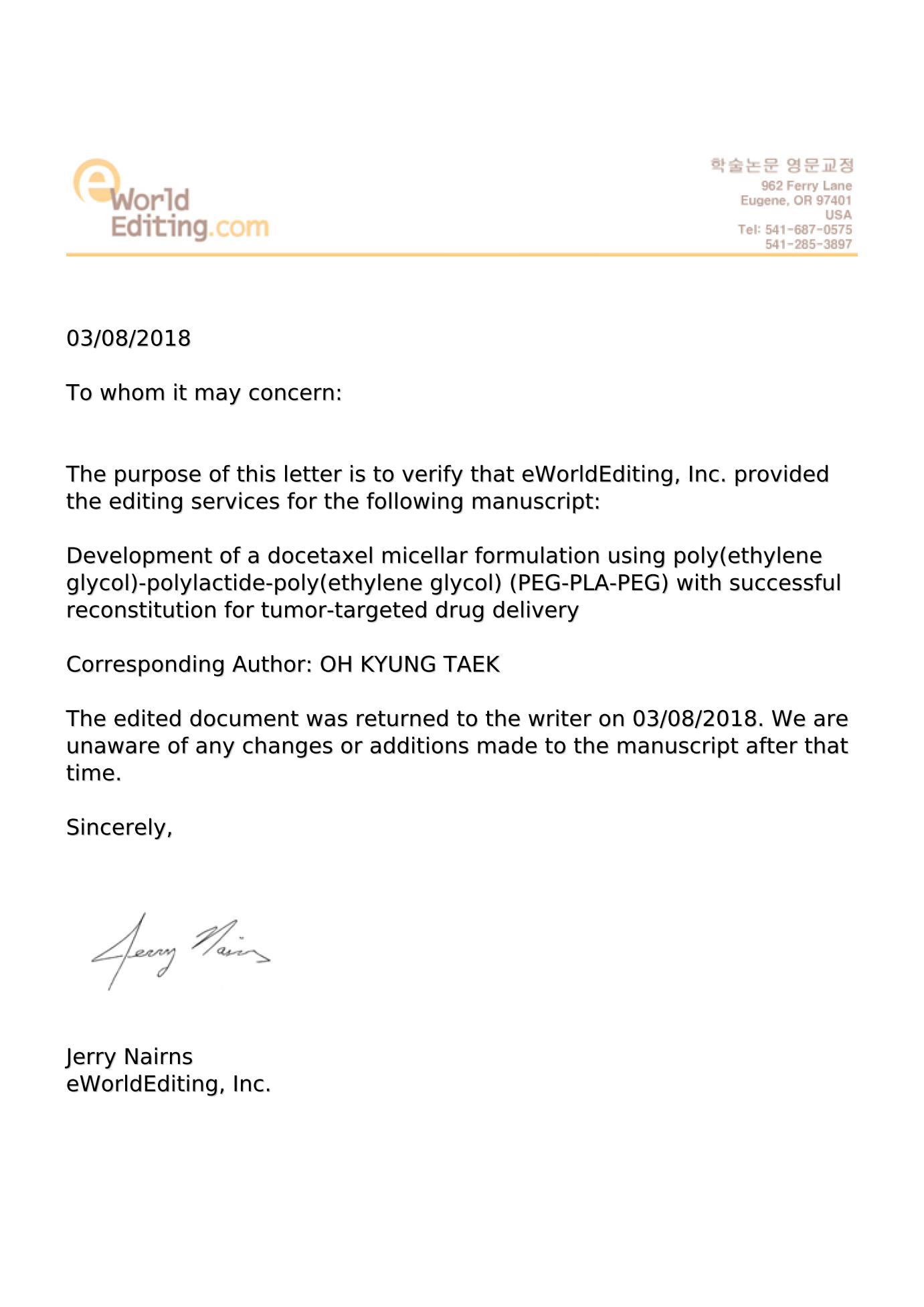

Supplement: Supplemental Material [file IDRD_A_1477865_SM6356.docx]
